# Dietary Fiber in Endometriosis: Mechanisms, Evidence, and Potential Clinical Benefits—Up-to-Date Review

**DOI:** 10.3390/nu18040690

**Published:** 2026-02-21

**Authors:** Justyna Czerniak, Michał Ciebiera, Natalia Zeber-Lubecka, Piotr Olcha

**Affiliations:** 1 Department of Human Nutrition, Institute of Human Nutrition Sciences, Warsaw University of Life Sciences (WULS-SGGW), Nowoursynowska 166, 02-787 Warsaw, Poland; 2 Warsaw Institute of Women’s Health, 00-189 Warsaw, Poland; 3 Second Department of Obstetrics and Gynecology, Centre of Postgraduate Medical Education, 01-813 Warsaw, Poland; 4 Department of Gastroenterology, Hepatology and Clinical Oncology, Centre of Postgraduate Medical Education, 02-781 Warsaw, Poland; 5 Department of Experimental Oncology, Laboratory of Cancer Metabolism, Maria Sklodowska-Curie National Research Institute of Oncology, 02-781 Warsaw, Poland; 6 Department of Gynecology and Gynecological Endocrinology, Medical University of Lublin, Aleje Racławickie 23, 20-049 Lublin, Poland

**Keywords:** dietary fiber, endometriosis, estrogen metabolism, inflammation, microbiota, women’s health

## Abstract

**Background:** Endometriosis is a chronic, estrogen-dependent inflammatory disorder. It is associated with hormonal dysregulation, pelvic pain, and a reduced quality of life. Dietary factors may influence disease development and symptom severity. **Objective:** This article aims to summarize current evidence on the role of dietary fiber in endometriosis and to discuss the underlying biological mechanisms and clinical implications. **Methods:** A narrative review of the literature was conducted using peer-reviewed original research articles and review papers published in English. Studies examining dietary fiber intake, fiber-rich dietary patterns, and their associations with endometriosis risk, symptoms, estrogen metabolism, gut microbiota, and inflammation. **Results:** Observational studies suggest that higher dietary fiber intake, particularly from fruits, vegetables, and whole grains, may be associated with a reduced risk of endometriosis. Interventional evidence remains limited. However, available studies indicate that fiber-rich dietary patterns may contribute to pain reduction and improvements in quality of life. **Conclusions:** Current evidence suggests that dietary fiber may play a supportive role in the prevention and management of endometriosis through multiple interconnected biological pathways. Nevertheless, the limited number of high-quality interventional studies highlights the need for further well-designed clinical trials to clarify the therapeutic potential of fiber-rich diets as an adjunct to standard endometriosis care.

## 1. Introduction

Endometriosis is defined by the presence of endometrial-like glands and/or stroma growing outside the uterus, presenting as a complex, chronic, and systemic inflammatory gynecological disorder [1]. This condition affects approximately 190 million girls and women worldwide [2]. It often leads to severe symptoms, including chronic pelvic pain, dysmenorrhea (painful menstruation), dyspareunia (painful intercourse), and infertility [3]. Given the frequency of symptoms, endometriosis significantly impairs the physical, mental, and social well-being of the affected women [4]. Additionally, endometriosis is frequently diagnosed with a considerable delay, resulting from both patient-related factors (such as a younger age at symptom onset and delayed healthcare seeking) and physician-related factors, including symptom normalization and limited awareness in primary care. These collectively prolong the time a patient suffers from the disease and delay comprehensive management [5]. Endometriosis is recognized as an estrogen-dependent disease. Therefore, factors modulating estrogen activity, inflammation, and immune dysfunction are central to its pathogenesis [6,7,8]. While current treatments focus on the surgical removal of lesions and chronic medical management (e.g., hormonal therapy), these approaches are not universally effective and may lead to recurrence [2]. There is a growing body of evidence indicating that dietary and nutritional interventions can modulate these underlying pathophysiological processes, specifically inflammation, estrogen pathways, and the gut microbiome [2,9,10,11]. Consequently, noninvasive nutritional strategies are emerging as essential adjunct interventions. Dietary fiber, a key component of healthy, plant-based diets, is of particular interest due to its documented ability to modulate systemic metabolic and hormonal balance [12,13]. This review synthesizes the current literature regarding dietary fiber, focusing on its proposed biological mechanisms in endometriosis and evaluating the existing clinical evidence from observational and interventional studies.

## 2. Materials and Methods

This article presents an updated review of publications on dietary fiber and its potential role in the management of endometriosis. A literature search for this narrative review was conducted in online databases (PubMed, Scopus, and Web of Science). The search included articles published until November 2025, using the following terms: “dietary fiber”, “fiber intake”, “endometriosis”, “estrogen metabolism”, “gut microbiota”, “inflammation”, “oxidative stress”, and “women’s health”.

The review included original research articles, such as randomized and non-randomized clinical trials, prospective observational studies, retrospective cohort studies, and case–control studies, as well as relevant review articles providing mechanistic or clinical context. Articles were considered eligible if they directly addressed the relationship between dietary fiber, fiber-rich dietary patterns, or specific fiber fractions and endometriosis-related outcomes, including disease risk, symptom severity, hormonal regulation, inflammatory pathways, oxidative stress, or gut microbiota composition. Only full-length articles published in English were included. Studies lacking a clear dietary exposure assessment or relevant endometriosis-related outcomes were excluded.

The identified evidence was critically analyzed to identify areas of consensus as well as inconsistencies across studies. When findings were conflicting, a greater emphasis was placed on studies with larger sample sizes, more robust study designs, and clearer outcome definitions. Given the heterogeneity of study designs, exposures, and endpoints, a narrative approach was adopted.

## 3. Fiber and Its Food Sources

Dietary fiber is generally defined as the edible parts of plants, or similar carbohydrates that are resistant to digestion and absorption in the small intestine [14]. More specifically, dietary fiber consists of food components, mainly found in cereals, fruits, and vegetables, that are resistant to human digestive enzymes. Traditionally, dietary fiber was defined as the portion of plant-based foods that is resistant to digestion, including polysaccharides and lignin. This definition was then expanded to include oligosaccharides such as inulin and resistant starches. Dietary fiber components that resist digestion in the small intestine are subsequently digested or partially fermented in the large intestine [15,16].

### 3.1. Classification and Components of Dietary Fiber

Dietary fiber may be divided into multiple fractions. The most accepted and common classification divides dietary fiber into soluble and insoluble fractions based on their chemical structure, water solubility, and functional properties within the gastrointestinal tract [14,16]. Soluble dietary fiber (SDF) dissolves in water to form viscous, gel-like solutions. These fibers are not digested in the small intestine but undergo extensive fermentation by colonic microbiota, producing short-chain fatty acids (SCFAs) that contribute to numerous physiological benefits. SDF includes compounds such as pectins, gum arabic, gums, inulin, galactomannans, and β-glucans. Insoluble dietary fiber (IDF) does not dissolve in water and does not form viscous solutions. These fibers are only minimally fermented in the colon and primarily contribute to stool bulk and intestinal transit. IDF consists mainly of lignin, cellulose, and certain hemicelluloses [17].

According to the Cereals & Grains Association (formerly the American Association of Cereal Chemists) [18], dietary fiber encompasses a broad range of structurally distinct components that extend beyond the soluble–insoluble dichotomy. These include:

- Non-starch polysaccharides and oligosaccharides: cellulose, hemicellulose, arabinoxylans, arabinogalactans, polyfructoses, inulin, oligofructans, galacto-oligosaccharides, gums, mucilages, and pectins.
- Carbohydrate analogs: indigestible dextrins, resistant maltodextrins, resistant potato dextrins, synthesized carbohydrate compounds, polydextrose, methyl cellulose, hydroxypropylmethyl cellulose, and resistant starches.
- Lignin and associated substances: waxes, phytate, cutin, saponins, suberin, and tannins, which are bound to or associated with the non-starch polysaccharide and lignin complex.

### 3.2. Dietary Sources of Fiber

Dietary fiber is an abundant component of plant foods, predominantly found in whole grains, vegetables, fruits, legumes, and nuts [19]. Most fiber-containing foods contain a blend of both types, commonly providing approximately one-third of soluble and two-thirds of insoluble fiber (Table 1). In many European countries, cereal-based products, particularly bread, are the primary sources of dietary fiber in the habitual diet [20]. However, this pattern contrasts with contemporary Westernized dietary habits, which are often characterized by reduced fiber intake due to the high consumption of ultra-processed foods. Such products generally contain markedly lower fiber levels compared to meals prepared from minimally processed or raw ingredients [21].

It is worth noting the structural and functional complexity of dietary fiber, thereby highlighting its diverse physiological effects. Understanding the distinctions among fiber fractions is essential for evaluating their specific roles in human health and for guiding dietary recommendations.

## 4. Biological Mechanisms of Dietary Fiber in Endometriosis

Dietary fiber may influence endometriosis through several biological pathways, including estrogen metabolism, gut microbiota, and inflammatory signaling. Each pathway reflects a distinct biological process: hormonal regulation, host-microbe interactions, and immune-mediated responses that may contribute to the pathophysiology of endometriosis in an independent or synergistic manner.

### 4.1. Estrogen Metabolism

Estrogens are key steroid hormones regulating the function of the female reproductive system. Estrogens synthesized in the ovaries are metabolized in the liver, where they are conjugated and released with the bile into the intestines. There, they may undergo deconjugation by intestinal microbiota enzymes and be subsequently reabsorbed into the circulation. The phenomenon is referred to as the enterohepatic circulation of estrogens. This process significantly influences the level of active estrogens in premenopausal women, determining the degree of proliferative stimulation of target tissues, including the endometrium [23,24].

Endometriosis is an estrogen-dependent disease in which the ectopic endometrial tissue exhibits sensitivity to estrogens and responds to them by proliferating and enhancing the inflammatory response from which it is inseparable [25]. The condition mainly occurs during the reproductive period, which is due to the hormonal activity of the ovaries. Numerous publications emphasized the role of estrogens in maintaining the growth of endometrial foci, thus intensifying angiogenesis and pain signaling in patients with endometriosis [26]. Higher estrogen exposure, resulting from frequent ovulation cycles, earlier menarche, and shorter menstrual cycles, was identified as a significant risk factor for the development of endometriosis, as indicated by epidemiological data included in reviews concerning the condition [27].

Some endometriosis hormone therapies aim to reduce the availability of estrogens—either by inhibiting ovulation or by lowering the bioavailable fraction of hormones. Researchers also indicated the possible therapeutic potential of nutritional interventions, e.g., increasing fiber intake [10]. Dietary fiber modulates estrogen metabolism mainly by inhibiting the enterohepatic circulation of estrogens, as mentioned above. It binds these hormones in the intestine, increasing their excretion with feces and reducing their reabsorption into the bloodstream [9,28]. A clinical trial conducted in premenopausal women showed that an increase in the supply of fiber resulted in a significant reduction in estrogen concentrations and was associated with anovulation [29]. Collectively, the mechanisms of these relationships probably include a decrease in the enterohepatic circulation of estrogens, a reduction in the free estradiol fraction, the modulation of the intestinal microbiota, and a reduction in the activity of β-glucuronidase, as well as effects that attenuate local inflammation in the ectopic endometrial environment.

Numerous analyses were conducted in this area. A similar effect was obtained under conditions of a strictly controlled dietary setting. The use of high-fiber nutrition led to estradiol and estrone reduction and to an extension of the follicular phase and the overall cycle, which might ultimately be associated with a lower total tissue exposure to estrogens [30]. Furthermore, a population-based BioCycle study revealed that higher fiber intake was associated with lower concentrations of steroid hormones during the menstrual cycle. However, it might simultaneously increase the risk of anovulatory cycles in healthy women, which confirms the significant impact of diet on the hypothalamic–pituitary–ovarian axis [29]. These findings suggest that a high-fiber diet is significantly associated with decreased hormone concentrations and a higher probability of anovulation.

Epidemiological data on endometriosis indicated that higher fiber intake reduced the risk of the disease. After accounting for confounding factors, higher fiber intake reduced the likelihood of endometriosis by over 40% (OR 0.588; *p* = 0.041) [12].

Given the above data, it is reasonable to conclude that estrogens are a key factor driving endometriosis, and dietary fiber may act as a natural regulator of their metabolism and bioavailability.

The role of estrogens in the pathogenesis and progression of endometriosis is well established and supported by a variety of experimental, clinical, and epidemiological evidence. We already know that the physiological relevance of the enterohepatic circulation in regulating circulating estrogen concentrations is clearly documented. Controlled dietary interventions and metabolic studies consistently demonstrate that increased dietary fiber intake enhances fecal estrogen excretion and reduces circulating estrogen levels, in some cases affecting ovulatory function.

Conversely, some mechanisms remain emerging or hypothetical in the context of endometriosis. Although the modulation of gut microbiota composition, the reduction in β-glucuronidase activity, and the anti-inflammatory effects of fiber-derived metabolites represent compelling pathways, direct causal evidence linking these mechanisms to clinically meaningful changes in endometriosis progression is still limited. Similarly, while observational studies suggest a protective association between higher fiber intake and a reduced disease risk, causality cannot be inferred from all analyses.

### 4.2. Gut Microbiota and the Estrobolome

The estrobolome should be conceptualized not as a single enzymatic shortcut but as a dynamic, diet-sensitive interface that integrates microbial enzymatic capacity, host enterohepatic circulation, immune tone, and barrier integrity [31]. Its functional output, i.e., the amount of bioactive estrogen that re-enters the systemic circulation, is an emergent property of this network rather than one dictated by a linear pathway [31,32]. Classic narratives emphasized that gut bacterial β-glucuronidase (GUS) deconjugated biliary estrogen glucuronides in the intestine, enabling reabsorption and raising systemic estrogen [33]. While correct, this view is reductionist. Estrobolome activity is contingent upon substrate supply (hepatic conjugates entering the gut), microbial ecology (which taxa carry and express distinct GUS isozymes), and co-metabolic networks that reshape epithelial transporters, tight junctions, and mucosal immune signaling, each of which modifies the enterohepatic gatekeeping of estrogen [31,34]. Evidence of endometriosis remains a signal embedded in noise [34,35,36]. Multiple human studies and syntheses suggested microbiota alterations in women with endometriosis. However, taxonomic findings were heterogeneous and often attenuated after multiple-testing correction [36,37]. This inconsistency argues for shifting focus from taxonomy to function—enzymes and metabolites that directly influence estrogen metabolism [35,38]. Still, targeted estrobolome readouts remain intriguing: a 2023 case-control study demonstrated the enrichment of *Erysipelotrichia* and higher fecal estrogen metabolites in endometriosis without intergroup differences in total β-glucuronidase activity, underscoring a pathway-level nuance rather than the monotonic “high GUS = high risk” paradigm [35].

Dietary fiber emerges as a systems modulator rather than a mere substrate. Fermentable fibers increase microbial SCFA production (acetate, propionate, butyrate), which strengthens the epithelial barrier and modulates host signaling via FFAR2, FFAR3, and GPR109A, partly through HDAC inhibition [39,40,41]. Recent reviews have extended these SCFA-mediated effects to gynecological contexts, including endometriosis [42,43,44,45].

Mucin-degrading taxa, particularly *Akkermansia muciniphila* and selected *Bacteroides* spp., add another layer of control by dynamically modulating mucus thickness, a physical determinant of epithelial access for both microbial enzymes and estrogen conjugates [46,47,48]. Thinner mucus may increase the effective contact time between luminal conjugates and the epithelial surface, potentially facilitating reabsorption if barrier tight junctions remain permissive [49]. The gut microbiota remodels primary into secondary bile acids (BAs). They signal through FXR and TGR5 to tighten epithelial junctions and repress NF-κB or NLRP3 activation, thereby lowering mucosal inflammatory tone and preserving barrier function [50,51,52]. Dysbiosis can shift the composition of the BA pool, recalibrating these signaling pathways and potentially altering the very conditions that favor estrogen reabsorption across the gut wall. Importantly, BAs interact with transporters such as ASBT (*SLC10A2*) in the ileum. Both BA and SCFA can modulate transporter expression, primarily through epigenetic and FFAR-mediated signaling, adding another layer of control over enterohepatic estrogen gatekeeping [53,54,55,56]. Framing the estrobolome as “estrogen-BA crosstalk” thus provides a more complete model than the GUS-centric one [31].

Emerging evidence suggests that the gut microbiota may act as a contextual buffer rather than a simple amplifier of estrogen exposure. Mechanistic studies indicated that the deconjugation and reabsorption of estrogen conjugates depended not only on microbial enzyme activity but also on barrier integrity and intestinal transit time, which governed mucosal contact [57]. Microbiota-derived fermentation products, particularly butyrate, were found to strengthen epithelial tight junctions and reduce permeability, potentially limiting excessive estrogen reabsorption even when β-glucuronidase was active [58]. Conversely, in low-estrogen states such as postmenopause, reduced conjugate supply and altered microbial diversity might lower reactivation capacity [57,59]. Dietary strategies that promote SCFA production or support BA signaling could enhance mucosal resilience and contribute to estrogen homeostasis, although direct clinical evidence remains limited [60]. This buffering model aligns with the immunologic bridge to lesion biology in endometriosis. Endometriosis is estrogen-dependent and immune-mediated, characterized by a Th17/Treg imbalance and heightened innate activation. Butyrate promotes Treg programs, reshapes epithelial and myeloid signaling, and can rebalance Th17/Treg axes [61,62]. Likewise, secondary BAs modulate barrier and inflammasome pathways [53]. Conceptually, positioning dietary fiber as an immune rheostat, rather than merely a prebiotic substrate, is congruent with this pathophysiology and with the observed links between gut inflammation, barrier leakage, and peripheral aromatase drive [63]. Butyrogenic Firmicutes, such as *Faecalibacterium prausnitzii*, *Roseburia*, and *Eubacterium* spp., were found to be central to SCFA production, supporting epithelial barrier integrity and modulating the immune tone [64,65]. Certain *Bacteroides* and *Clostridium* clusters harbored diverse GUS isozymes implicated in estrogen deconjugation [33]. *Lactobacilli*, often reduced in dysbiotic states but variably associated with endometriosis, contributed to mucosal homeostasis primarily through lactic-acid signaling and BA remodeling rather than GUS activity [66,67]. A smaller but mechanistically relevant group of microbial enzymes comprised hydroxysteroid dehydrogenases (HSDHs), which catalyzed reversible interconversions between active and inactive steroid forms [68]. Several gut taxa, notably *Clostridia* and *Eggerthella* spp., encoded 3α-, 3β-, and 17β-HSDH homologs capable of modifying steroid backbones, potentially influencing downstream conjugation patterns [69]. Current evidence for these activities primarily derives from studies on BA and androgen metabolism. However, metagenomic reconstructions suggested that analogous pathways might also act on estrogen intermediates [68]. Although direct functional validation in estrogen metabolism remains limited, these findings add complexity to the estrobolome and position microbial HSDHs as plausible contributors to local steroid signaling within the gut.

Several mechanistic links described in this system framework remain better supported at the level of plausibility than direct empirical demonstration, and clarifying these boundaries strengthens the overall interpretation. First, while the inhibition of class I HDACs by butyrate is well documented, its effects extend beyond HDAC1 and HDAC3 to include HDAC2 and HDAC8. Thus, references to specific isoforms should be understood as representative rather than exhaustive [70]. Second, the SCFA-mediated modulation of intestinal transporter expression, including estrogen-relevant solute carriers, was reported in cell and animal models, but human evidence is still sparse, making it a promising, yet incompletely validated, regulatory axis [71]. Third, the proposition that BAs modulate enterohepatic estrogen gatekeeping is mechanistically coherent, given their effects on barrier integrity, immune tone, and transporter expression, but direct studies quantifying how BA signaling alters estrogen reabsorption in vivo are lacking [72,73]. Finally, the suggestion that the SCFA-induced tightening of the epithelial barrier may constrain estrogen reactivation and reabsorption is conceptually attractive and consistent with the known biophysics of mucosal permeability, though it has not yet been empirically measured in humans [74]. These caveats do not contradict the broader system model. Rather, they delineate which components are firmly evidence-based and which represent testable hypotheses that should guide the next generation of mechanistically anchored studies.

A mechanism-focused approach, addressing estrobolome function, SCFA and BA signaling, and barrier integrity, appears the most promising for identifying testable targets such as dietary fiber [75]. These insights help explain inconsistent taxonomy-level findings and underscore the value of functional biomarkers and diet-based strategies for future research. While several mechanisms are well supported experimentally, others remain hypothetical and require targeted human studies. Overall, the estrobolome should be interpreted as a network-level regulator, with dietary fiber acting as a system-level modulator rather than a direct estrogen-metabolizing agent.

### 4.3. Inflammation and Oxidative Stress

Endometriosis is a chronic, estrogen-dependent inflammatory disease in which the impairment of the immune response and increased oxidative stress play a key role. The peritoneal fluid and endometrial foci exhibit enhanced macrophage activity, elevated levels of proinflammatory cytokines such as IL-1β, IL-6, and TNF-α, and overproduction of reactive oxygen species (ROS), which may be collectively defined as proangiogenic factors promoting the proliferation of ectopic tissue and the persistence of chronic inflammation [76,77].

A growing body of evidence suggests that dietary factors, including dietary fiber intake, may modulate these mechanisms by influencing the gut microbiota, the intestinal barrier, and estrogen metabolism [78].

Dietary fiber, particularly its fermentable fractions (e.g., inulin, fructooligosaccharides, and beta-glucans), is a primary substrate for intestinal bacteria producing SCFAs, including butyrate, propionate, and acetate. These microbial metabolites exert well-documented anti-inflammatory effects through the inhibition of the activation of the transcription factor NF-κB, the modulation of NLRP3 inflammasome activity, and the promotion of the differentiation of regulatory T cells (Treg) [79]. In endometriosis, disturbances in gut microbiota composition (dysbiosis) are observed, which may trigger chronic systemic and local inflammation. A low-fiber diet is associated with decreased production of SCFAs and increased intestinal barrier permeability, which facilitates the translocation of lipopolysaccharides (LPSs) and the subsequent activation of the inflammatory response [78,80]. Therefore, increasing fiber intake may indirectly attenuate inflammation by improving intestinal barrier integrity and reducing endotoxemia.

Oxidative stress is a critical component in the pathophysiology of endometriosis and is closely associated with the inflammatory process. Excess reactive oxygen species (ROS) promote lipid peroxidation, DNA damage, and the activation of proinflammatory pathways, thereby establishing a positive feedback loop between inflammation and oxidative stress [76,77]. The effect of dietary fiber on oxidative stress is indirect and is primarily mediated through its inflammation-reducing effects and the modulation of the gut microbiota. SCFAs produced via fermentation, particularly butyrate, exert cytoprotective effects and may upregulate the expression of antioxidant enzymes, including superoxide dismutase (SOD) and catalase [58,81]. Additionally, enhanced intestinal barrier integrity reduces exposure to pro-oxidative bacterial factors, potentially contributing to a lower overall oxidative burden.

Endometriosis is highly dependent on estrogens, which may exacerbate inflammatory processes and oxidative stress in the target tissues. Dietary fiber exerts a beneficial effect on the enterohepatic circulation of estrogens by increasing their fecal excretion and modulating gut microbiota activity, including the estrobolome, i.e., a consortium of bacteria capable of deconjugating estrogens [80,82,83]. Recent epidemiological studies and Mendelian randomization analyses have indicated that a higher fiber intake may be associated with a reduced risk of endometriosis, potentially by reducing estrogen bioavailability and an indirect limitation of the proinflammatory progression of the disease [12,84].

**Figure 1:**
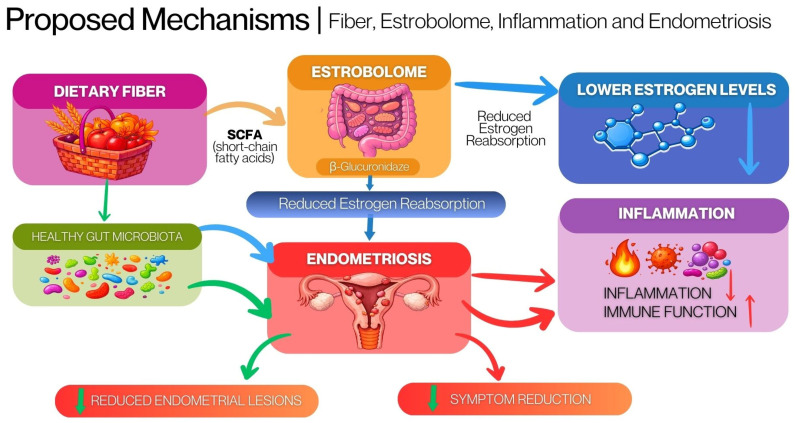
Proposed mechanisms linking dietary fiber, the estrobolome, inflammation, and endometriosis. **Blue arrows** indicate effects related to estrogen metabolism and reduced estrogen reabsorption, **red arrows** represent inflammatory and immune-related pathways, and **green arrows** denote gut microbiota-mediated effects. Downward arrows indicate a reduction in the respective process or outcome. Created in Canva by the authors.

Observational studies suggested a potential protective association between fiber intake and the risk of developing endometriosis. However, the interpretation of their findings is limited by potential errors in dietary assessment and the concurrent presence of other dietary components with anti-inflammatory and antioxidant properties [12,83]. Current systematic reviews highlight the need for randomized interventional studies focused on a definitive evaluation of the effects of fiber quantity and type on inflammatory markers, oxidative stress, and the clinical symptoms of endometriosis [77,83]. These interacting pathways are summarized in Figure 1.

## 5. Clinical Evidence on Dietary Fiber and Endometriosis

### 5.1. Observational Studies

Multiple observational studies investigated the relationship between dietary fiber intake and the risk of endometriosis, generally suggesting an inverse association. However, some authors reported conflicting findings. Cross-sectional analyses of U.S. survey data linked higher total fiber intake to a lower prevalence of endometriosis. For example, Zheng et al. [12] (NHANES 1999–2006, *n* = 2840) found that, after extensive multivariate adjustment, women in the highest quartile of fiber intake (Q4: 27.95–111.40 g/day) had significantly lower odds of endometriosis: each 1 g/day increase in fiber was associated with a 41.2% reduction in the prevalence (OR 0.588; *p* = 0.041). Similarly, a dose–response trend was demonstrated for fiber (*p* = 0.037), and smoothed curve analysis confirmed a decrease in endometriosis risk at higher intakes. However, a recent reanalysis of the same NHANES study [13] but with a higher number of participants (n = 4453) showed a positive association: women in the highest fiber quartile (Q4: 17.6–128.3 g/day) had increased odds of self-reported endometriosis (OR 1.73, *p* = 0.011), and the overall trend per gram of fiber was significant (*p* = 0.034). Chen et al. [85] analyzed the same study group and found an association between the dietary inflammatory index (DII) and endometriosis. They reported that women with endometriosis (n = 287) had a significantly lower mean fiber intake compared to the controls (13.0 vs. 14.6 g/day, *p* = 0.001).

Other studies consistently suggested that the protective effect of fiber in endometriosis was primarily driven by vegetable sources. In the Italian case–control studies summarized by Parazzini et al. [86] (504 laparoscopically confirmed cases and 504 controls), the strongest inverse association was observed specifically for green vegetables, with women in the highest tertile of intake exhibiting nearly a 70% decrease in the risk (OR 0.30, *p* = 0.0001) compared to those in the lowest tertile. Such a pronounced effect was not mirrored by other fiber sources: while fresh fruit intake showed a moderate protective trend (OR 0.6, *p* = 0.0001), whole grain products were not associated with the risk (OR 1.0, not significant). Similarly, a 2019 Iranian case–control study [87] (78 cases, 78 controls) revealed that higher fruit (Q4 OR 0.29; *p* = 0.10) and vegetable (Q4 OR 0.38; P-trend = 0.25) consumption was associated with a lower risk of endometriosis. An increased consumption of potatoes, one of the starchy vegetables, was associated with a reduced risk of endometriosis (Q4 OR 0.40; P-trend = 0.11), but a substantial increase in the risk of endometriosis was noted for fried potatoes (Q3 OR 4.13; P-trend = 0.19). Women with the highest intake of soluble fiber had 67% lower odds of endometriosis compared to those in the lowest quartile (OR 0.33). Simultaneously, a more modest but still significant reduction was observed for insoluble fiber (OR 0.76). A significant trend across quartiles was observed for both fiber types (P-trend = 0.04). Total fiber exerted no significant effect in this analysis (*p* > 0.05). Trabert et al. [88] (284 cases, 660 controls) reported no significant association between total vegetable intake and endometriosis and, in fact, observed higher odds with greater fruit intake (OR 1.5 for >2 vs. ≤1 servings/day). By contrast, Savaris et al. [89] conducted a study in Brazil (25 cases, 20 controls) and observed that women with endometriosis actually had a higher total fiber intake than the controls (*p* < 0.05). In both groups, fiber intake was lower than the recommended 25 g/day.

Prospective cohort studies are more limited, but they largely support a protective role for fiber-rich foods. In the Nurses’ Health Study II [90], 70,835 premenopausal women free of endometriosis at baseline were followed up from 1991 to 2013 with repeated food frequency questionnaires and laparoscopic confirmation of new cases. Women consuming ≥ 1 serving of citrus fruits/day had a 22% lower endometriosis risk (RR 0.78; P-trend = 0.004) compared to those consuming < 1 serving/week. No significant association was observed for total vegetable intake. More detailed analyses were conducted by Schwartz et al. [91] using the same data (Nurses’ Health Study II) collected from 81,961 premenopausal women. The study demonstrated that the relationship between fiber and endometriosis might strongly depend on the fiber source: higher intakes of vegetable and cruciferous vegetable fiber were associated with a modestly increased risk (RR 1.13 and 1.17, respectively), whereas fruit-derived fiber showed an inverse association (RR 0.90). No associations were observed for cereal or legume fiber. Additionally, a higher dietary glycemic index was associated with an increased risk (the highest quintile, RR 1.12).

### 5.2. Interventional Studies

Although observational evidence suggested potential benefits of dietary modification in the management of endometriosis, interventional studies remain limited [2,11]. To date, no randomized controlled trials (RCTs) have identified fiber as a single dietary factor in endometriosis. Available trials, primarily focused on Mediterranean-style dietary patterns, consistently demonstrated reductions in symptoms and, consequently, improvements in quality of life.

Ott et al. [92] conducted a prospective experimental study in 68 women with laparoscopically diagnosed endometriosis, evaluating the effect of adopting the Mediterranean diet (MD) on pain severity. The intervention required patients to adhere to an MD regimen for 5 months, emphasizing foods rich in fiber, such as fresh vegetables and fruit, and wholemeal products, while recommending the avoidance of red meat, sweets, and sugary drinks. Significant improvements were observed across all measured symptoms. In the intention-to-treat analysis, general pain decreased from 4.2 ± 2.5 to 2.5 ± 2.4 (*p* = 0.003), while the general condition improved from 6.4 ± 1.9 to 8.2 ± 1.8 (*p* = 0.005). Specific pain domains showed similar benefits: dysmenorrhea decreased from 4.6 ± 2.8 to 2.9 ± 2.5, dyspareunia from 1.8 ± 2.2 to 0.9 ± 1.3, and dyschezia from 2.9 ± 3.1 to 2.0 ± 2.5 (all *p* < 0.05). A more recent prospective 6-month intervention by Cirillo et al. [93] was conducted to evaluate 35 women with confirmed endometriosis, investigating the effect of the MD over a 6-month period (T1: 3 months; T2: 6 months). The score of adherence to the MD (range 0–18) significantly increased at both time points (*p* < 0.001). The improvement was marked by the enhanced consumption of fruits (*p* = 0.001 at T1; *p* = 0.003 at T2), vegetables (*p* = 0.003 at T1 and T2), legumes (*p* = 0.003 at T1), and cereals (*p* < 0.001 at T1; *p* = 0.007 at T2). Correspondingly, significant reductions were observed for several pain parameters at 3 months, especially for dyschezia (*p* < 0.001), but also for dyspareunia (*p* = 0.04), non-menstrual pelvic pain (*p* = 0.06), and dysuria (*p* = 0.04). At 6 months, improvements deepened for dyspareunia (*p* = 0.002) and dyschezia (*p* < 0.001). The study also identified mechanistic correlates: higher dietary adherence was associated with significantly lower reactive oxygen species (ROS) production at T2 (neutrophil-ROS: rho = −0.55, *p* = 0.03; monocyte-ROS: rho = −0.66, *p* = 0.007; lymphocyte-ROS: rho = −0.55, *p* = 0.03) and improved folate levels (folate increased from 5.6 to 7.1 ng/mL, *p* = 0.006).

Findings from the recent scoping review by De Araugo et al. [94] highlight that, despite the frequent use of dietary strategies by individuals with endometriosis, high-quality interventional evidence remains scarce. This review (n = 13 studies, 7 RCTs) assessed the effect of nutritional interventions on confirmed endometriosis. However, only 5 studies involved dietary interventions, while the remaining ones tested supplements or combined approaches. While some interventions, such as low FODMAP diets, Mediterranean-style dietary patterns, or nickel-restricted diets, demonstrated reductions in selected pain symptoms or improvements in gastrointestinal outcomes, these effects were not consistently replicated across studies. The overall quality of evidence was poor, and high-quality RCTs are urgently needed to confirm the therapeutic role of diet in endometriosis management. The key observational and interventional evidence summarized in this section is compiled in Table 2.

## 6. Discussion

The findings of this review provide growing, yet heterogeneous, evidence suggesting that dietary fiber may influence the risk and symptoms of endometriosis. The biological rationale for the benefits of fiber is primarily centered on its potential to regulate estrogen metabolism and attenuate systemic inflammation.

### 6.1. Observational Evidence

The authors of several epidemiological studies examined whether diets rich in plant fiber were associated with a lower incidence of endometriosis. The results were mixed. Observational studies based on the U.S. population (NHANES) identified strong inverse associations between fiber intake and endometriosis risk. Zheng et al. [12] demonstrated that women in the highest quartile of fiber intake had a reduction in disease prevalence of over 40%. Li and Zhang [95] further noted that this protective effect was most pronounced in normal-weight women (BMI < 25 kg/m^2^), suggesting that excess adipose tissue in obese individuals might produce sufficient endogenous estrogen and proinflammatory cytokines to overshadow the benefits of fiber. Conversely, studies such as those by Harris et al. [90] and Schwartz et al. [91] indicated that the source of fiber was essential. While fiber from fruits (especially citrus) was associated with a reduced risk, fiber from cruciferous vegetables had a positive correlation with endometriosis diagnosis in some analyses. This may result from diagnostic bias, as cruciferous vegetables are high in FODMAPs, which may exacerbate gastrointestinal symptoms such as bloating and pain. Women experiencing such symptoms may be more likely to seek medical evaluation, leading to the discovery of existing but otherwise silent endometriosis. As over 90% of patients with endometriosis experience gastrointestinal symptoms, fiber-rich interventions are crucial for improving the quality of life [96,97].

### 6.2. Interventional Evidence

In contrast, interventional studies evaluating the effects of dietary modification in endometriosis are scarce and largely indirect. To date, no randomized controlled trials have specifically isolated dietary fiber as a single intervention. The existing trials have primarily examined fiber-rich dietary patterns, such as the Mediterranean diet, which involve concurrent changes in multiple nutrients with inflammation-reducing and antioxidant properties. Research on the Mediterranean diet showed significant reductions in non-menstrual pelvic pain, dyspareunia, and, most notably, dyschezia (painful defecation) [93]. These clinical benefits are attributed to the shortened intestinal transit time and the production of SCFAs, such as butyrate. These microbial metabolites strengthen the gut barrier and act as anti-inflammatory agents by inhibiting NF-*κ*B signaling pathways. While these interventions consistently demonstrate improvements in pain-related outcomes and quality of life, they do not allow definitive conclusions regarding the independent effect of fiber intake.

Overall, the available evidence suggests that dietary fiber may influence endometriosis through interconnected hormonal, immunological, and gut-mediated pathways. By reducing the enterohepatic recirculation of estrogens, fiber intake may contribute to lower systemic estrogen exposure, a key factor in the pathophysiology of endometriosis. Concurrently, the fiber-driven modulation of the gut microbiota and an increased production of SCFAs, particularly butyrate, may attenuate inflammatory signaling and oxidative stress via mechanisms involving gut barrier integrity and immune regulation. These effects are especially relevant given the high prevalence of gastrointestinal symptoms in women with endometriosis and the emerging concept of gut–pelvic axis interactions. Importantly, the heterogeneity of study designs and dietary interventions suggests that the clinical impact of fiber likely depends on fiber type, source, and individual metabolic context, including body composition and baseline inflammatory status. While these mechanistic correlates provide biological plausibility, causal pathways remain to be confirmed in well-designed, fiber-specific interventional studies that incorporate objective hormonal, inflammatory, and microbiome-related biomarkers.

The interpretation of the above findings is tempered by substantial limitations. The primary limitation of current knowledge is the lack of randomized controlled trials that isolate dietary fiber as a variable independent of other factors. Furthermore, dietary studies inherently rely on self-reporting (FFQs), which is prone to recall error and misclassification. Case–control designs are especially susceptible to bias: women who know they have endometriosis may recall their diet differently than controls, and selection bias may arise in clinic-based recruitment. As noted by Ghasemisedaghat et al. [98] in their case–control analysis, “selection bias, measurement bias, and recall bias could lead to erroneous conclusions”. Cohort studies may be confounded by the overall lifestyle; for example, women who eat more fiber may also exercise more or exhibit other health behaviors. The diagnostic definition of endometriosis itself is heterogeneous across studies. However, some authors required surgical (laparoscopic) confirmation, while others accepted imaging or even symptom-based criteria. This variability in case ascertainment affects the prevalence and may dilute the strength of associations. Many interventions were short-lasting (3–6 months) and underpowered. As highlighted by Cirillo et al. [93], larger study populations and longer intervention durations (e.g., ≥12 months) were required to confirm those preliminary findings. In addition, interventional diets often changed multiple nutrients simultaneously (e.g., omega-3 fats, antioxidants, and fiber), making it difficult to isolate the specific role of fiber. Finally, few studies [89,93] incorporated objective biomarkers (e.g., serum estrogens, inflammatory markers, or microbiome sequencing); thus, our understanding of biological mechanisms is still indirect. This limitation is not unique to dietary fiber but reflects a broader challenge in nutritional and complementary research in endometriosis. A review of phytotherapeutic approaches indicated that, while biological mechanisms and preliminary findings were encouraging, further well-designed, adequately powered clinical trials were needed to substantiate their therapeutic potential [99].

## 7. Conclusions and Future Directions

In summary, dietary fiber is a promising non-pharmacological tool for the integrated management of endometriosis.

- Dietary fiber may reduce endometriosis risk and symptoms via the modulation of estrogen metabolism, the gut microbiota, and inflammation.
- From a clinical perspective, fiber-rich dietary patterns, particularly those emphasizing fruits and vegetables, appear to be a low-risk adjunct to standard endometriosis care.
- Evidence from interventional studies remains limited, and fiber-specific randomized controlled trials are scarce.
- Future research should clarify the roles of fiber type (soluble vs. insoluble), source, and dose, and assess long-term clinical and hormonal endpoints.

## Figures and Tables

**Table 1:** The dietary fiber content of selected seaweeds, fruits, vegetables, legumes, and cereals, expressed as a percentage of dry matter, as reported by Escrig and Muniz [22].

Food Source	Insoluble Fiber (%)	Soluble Fiber (%)	Total Fiber (%)
**Cereals/Grains**			
Rice	0.75	0.19	0.94
Whole corn	87.47	0.40	87.87
Whole wheat	41.59	2.87	44.46
**Fruits**			
Apricots	44.92	26.43	71.35
Apples	55.57	18.56	74.13
Peaches	39.53	27.30	66.83
**Vegetables**			
Brussels sprouts	30.23	6.16	36.39
Onions	13.32	3.59	16.89
Potatoes	4.85	2.14	6.99
**Legumes**			
Beans	25.64	10.85	36.49
Chickpeas	16.69	1.35	18.04
Whole soy	65.24	7.08	72.32
**Seaweeds**			
Hijiki	16.3	32.9	49.2
Nori	16.8	17.9	34.7
Wakame	5.3	30.0	35.3

**Table 2:** Summary of observational and interventional studies evaluating the association between dietary fiber intake, fiber-rich dietary patterns, and endometriosis risk or symptom severity.

Authors, Publications Years, Country	Type of Study	Number of Subjects	Endometriosis Outcome	Key Fiber-Related Dietary Factor	Key Finding
Zheng et al., 2024, U.S. [12]	cross-sectional	2840 women (NHANES)	Self-reported endometriosis prevalence	Total dietary fiber	Each 1 g/day increase in total fiber was associated with 41% lower odds of endometriosis.
Huang et al., 2025, U.S. [13]	cross-sectional	4453 women (NHANES)	Self-reported endometriosis prevalence	Total dietary fiber	Women in the highest fiber quartile had higher odds of endometriosis compared with the lowest quartile (OR 1.73).
Chen et al., 2025, U.S. [85]	cross-sectional	4149 women (NHANES)	Self-reported endometriosis prevalence	Dietary inflammatory index (DII)	Women in the highest DII quartile had higher odds of endometriosis compared with the lowest quartile (OR 1.40).
Parazzini et al., 2004, Italy [86]	case–control	504 cases/ 504 controls	Laparoscopically confirmed endometriosis	Vegetables	High intake of green vegetables was associated with a 70% lower risk of endometriosis.
Youseflu et al., 2019, Iran [87]	case–control	78 cases/ 78 controls	Laparoscopically confirmed endometriosis	Soluble/insoluble fiber	Higher soluble fiber intake was associated with 67% lower odds of endometriosis.
Trabert et al., 2011, U.S. [88]	case–control	284 cases/ 660 controls	Surgically confirmed endometriosis	Fruit	Higher fruit intake was associated with increased odds for endometriosis (>2 servings fruit/day vs. ≤1: OR 1.5).
Savaris et al., 2011, Brazil [89]	case–control	25 cases/ 20 controls	Laparoscopically confirmed endometriosis also by histology	Total dietary fiber	Women with endometriosis reported higher total fiber intake than controls, although intake was below recommendations in both groups.
Harris et al., 2018, U.S. [90]	cohort	70,835 women (Nurses’ Health Study II)	Incident laparoscopically confirmed endometriosis	Fruit	Consumption of ≥1 serving/day of citrus fruits was associated with a 22% lower risk of endometriosis.
Schwartz et al., 2022, U.S. [91]	cohort	81,961 women (Nurses’ Health Study II)	Incident laparoscopically confirmed endometriosis	Fiber by sources (fruit, vegetables)	Fruit-derived fiber was inversely associated with endometriosis risk, whereas vegetable fiber showed positive associations.
Ott et al., 2012, Austria [92]	interventional	68 women (diagnosed endometriosis)	Endometriosis-related pain degrees (Numeric Rating Scale)	Mediterranean-style diet (rich in vegetables, fruits, whole grains)	5-month Mediterranean diet led to significant improvements in overall pain, and reduced dysmenorrhea, dyspareunia, and dyschezia.
Cirillo et al., 2023, Italy [93]	interventional	35 women (diagnosed endometriosis	Endometriosis-related pain degrees (Visual Analogue Scale)	Mediterranean-style diet (rich in vegetables, fruits, whole grains)	6-month Mediterranean diet significantly reduced dyspareunia, non-menstrual pelvic pain, dysuria, and dyschezia at 3 and 6 months.

## Data Availability

Not applicable.

## Abbreviations

The following abbreviations are used in this manuscript:

ASBT	Apical Sodium-Dependent Bile Acid Transporter
BAs	Bile Acids
BMI	Body Mass Index
cAMP	Cyclic Adenosine Monophosphate
DII	Dietary Inflammatory Index
FFAR	Free Fatty Acid Receptor
FFQ	Food Frequency Questionnaire
FXR	Farnesoid X Receptor
GPR	G Protein-Coupled Receptor
GUS	β-Glucuronidase
HDAC	Histone Deacetylase
HSDH	Hydroxysteroid Dehydrogenase
IDF	Insoluble Dietary Fiber
IL	Interleukin
LPS	Lipopolysaccharide
MD	Mediterranean Diet
NF-κB	Nuclear Factor Kappa B
NHANES	National Health and Nutrition Examination Survey
NLRP3	NOD-, LRR-, and Pyrin Domain-Containing Protein 3
OR	Odds Ratio
RCT	Randomized Controlled Trial
ROS	Reactive Oxygen Species
RR	Relative Risk
SCFAs	Short-Chain Fatty Acids
SDF	Soluble Dietary Fiber
SLC10A2	Solute Carrier Family 10 Member 2
SOD	Superoxide Dismutase
TGR5	Takeda G Protein-Coupled Receptor 5
TNF-α	Tumor Necrosis Factor Alpha
Treg	Regulatory T Cell
